# Thermal Tolerance and Physiological Changes in Mud Crab, *Scylla paramamosain* Crablet at Different Water Temperatures

**DOI:** 10.3390/ani11041146

**Published:** 2021-04-16

**Authors:** Muhammad Nur Syafaat, Mohamad Nor Azra, Faridah Mohamad, Che Zulkifli Che-Ismail, Adnan Amin-Safwan, Mohammad Asmat-Ullah, Mohammad Syahnon, Azmie Ghazali, Ambok Bolong Abol-Munafi, Hongyu Ma, Mhd Ikhwanuddin

**Affiliations:** 1 Research Institute for Brackishwater Aquaculture and Fisheries Extension, Maros 90512, Indonesia; muhammad.nursyafaat@kkp.go.id; 2 Institute of Tropical Aquaculture and Fisheries, Universiti Malaysia Terengganu, Kuala Nerus 21030, Malaysia; azramn@umt.edu.my (M.N.A.); aminsafwan14@gmail.com (A.A.-S.); asmatumt@gmail.com (M.A.-U.); syahnon.m@umt.edu.my (M.S.); azmie@umt.edu.my (A.G.); munafi@umt.edu.my (A.B.A.-M.); 3 Faculty of Science and Marine Environment, Universiti Malaysia Terengganu, Kuala Nerus 21030, Malaysia; mfaridah@umt.edu.my; 4 Crustacean Aquaculture Research Division, Fisheries Research Institute, Pulau Sayak, Kota Kuala Muda 08500, Kedah, Malaysia; zulkif009@gmail.com; 5 STU-UMT Joint Shellfish Research Laboratory, Shantou University, Shantou 515063, China; mahy@stu.edu.cn; 6 Guangdong Provincial Key Laboratory of Marine Biotechnology, Shantou University, Shantou 515063, China

**Keywords:** aquaculture, environmental conditions, seed production, mud crab, *Scylla paramamosain*, nursery, megalopa, crablet

## Abstract

**Simple Summary:**

The nursery phase of mud crab (*Scylla* spp.)—from megalopa to several, further crablet stagesneeds to be carried out in optimal environmental conditions, until they reach a larger size suitable to being stocked to a pond. This study observed the behavioral thermoregulation of crablet instar phases, and determined the effects of several levels of water temperature on growth, survival, molting cycle, gill condition, and sex ratio in the nursery phase of *S. paramamosain*. In this study, optimal temperature for the long-term nursery phase of *S. paramamosain*, beginning with megalopa stage, was found to be 28 to 30 °C, with the possibility that water temperature could affect the sex ratio of mud crabs. Findings of this study could result in increased crablet production from hatcheries, and assist in realizing potential of monosex seed production of *S. paramamosain*, through temperature treatment.

**Abstract:**

This study was carried out to determine the physiological changes (survival, growth, molting cycle, sex differentiation, and gill condition) of mud crab, *Scylla paramamosain* crablet at different water temperatures of 24, 28 and 32 °C, and ambient temperature of 27 to 30 °C. Thermoregulatory behavior, represented by preferred temperature (29.83 ± SD 2.47 °C), critical thermal minimum (17.33 ± SD 0.58 °C), critical thermal maximum (40 ± SD 0.00 °C), and thermal tolerance interval (22.67 ± SD 0.58 °C), were checked for Crablet 1 stage only (with ambient temperature as acclimation temperature).Both low (24 °C) and high (32 °C) temperatures were associated with lower growth performance, and survival rate (*p* < 0.05), in comparison with both 28 °C and ambient temperature treatments.Male ratio at low temperaturetreatment (24 °C) was higher (80.09 ± SD 18.86%) than for other treatments (*p* < 0.05), observed as 44.81 ± D 10.50%, 41.94 ± SD 19.44%, and 76.30 ± SD 5.13% for 28 °C, 32 °C and ambient temperature treatments, respectively. However, there was no significant difference observed between 24 °C, 28 °C, and ambient temperature treatments. Anatomical alterations of gill lamellae of *S. paramamosain* crablet for both 32 °C, and 24 °C treatments, appeared thinner and paler than at both 28 °C, and ambient temperature treatments. Based on this study, temperature of 28 to 30 °C was recommended as the optimal temperature for the long-term nursery phase of *S. paramamosain*.

## 1. Introduction

*Scylla paramamosain* is one of four species of mud crabs from the genus *Scylla*, based on revision of Keenan et al. [1]. It is a large, portunid crab found along coasts of southern China [2,3], and many Indo-Pacific countries [2]. Each species of mud crab has its own distribution area, and *S. serrata* has the widest distribution area in comparison with other species [4]. Temperature is one of the factors that affects patterns of geographic distribution, growth, maturation, development, and abundance of ectothermic organisms [5,6,7,8,9,10]. Each organism will try to find a location that has an optimal temperature for itself, because the condition will support optimization of physiological and biochemical processes, that in turn provides maximal efficiency in energy use [11,12], to maximize its growth [13,14].

In aquaculture, it is very important to understand the thermoregulatory behavior of cultured organisms. Thermoregulatory behaviour is used to inform about the tolerance limits, and the preferred temperature of aquatic species [7], and there is a relationship between thermoregulatory behaviour and optimum temperature for several physiological functions, such as metabolism, reproduction, and growth [15]. Critical thermal limits of a species are determined through exposure to a rapid constant change of temperature (increase or decrease), until a predefined, sublethal, but near lethal temperature is reached [16]. The nervous system in this condition is perturbed as a consequence of ionic unbalance provoked by temperature [7], which typically causes locomotory activity to become disorganized, while the animal loses its ability to escape from conditions that will promptly lead to its death [8,17]. In the critical thermal limits experiment, lethal temperatures are estimated without actually killing the animal [16]. The difference between critical thermal maximum (CTmax) and critical thermal minimum (CTmin) mean values, defined as a thermal tolerance interval (TTI), can be used to indicate the extent of tolerance of a species to temperature change [7]. Paschke et al. clarified that behavioral thermoregulation can be adaptive in two complementary ways: (i) it can help an organism avoid extreme heat or cold that might be damaging or lethal, and (ii) it can increase the time an animal spends at optimal physiological temperatures [7].

Temperature is also known as the most important environmental, sex determining factor for aquatic species [18]. Some studies show that water temperature can affect the sex ratio of some fish species, as in tilapia [19,20,21], guppy [22], medaka [23], and european sea bass [24], and of some crustaceans namely *Porcellionides pruinosus* (Isopod) [25], *Tigriopus californicus* (Copepod) [26], and *Neocaridina davidi* (Caridea) [18]. Knowledge of the extent to which temperature affects sex ratios is relevant to gauge potential threats of rising temperature on fish populations [27]. To date, there is no research on the impact of water temperature on sex ratio of mud crab, genus *Scylla*. A study of the impact of water temperature on sex ratio of mud crab is expected to provide opportunities to produce monosex seeds, especially for aquaculture purposes.

Studies on the effect of temperature on the nursery phase of *S. paramamosain*, for long term periods, is still scarce. Previous research conducted by Gong et al. [2] only looked at the effect of temperature on crablets from Crablet 1 to Crablet 2 stages, and Ruscoe et al. [28] only from Crablet 2 to Crablet 4. On the other hand, the stocking of crablets directly to the pond, after molting from the megalopa phase, is considered to be prone to risk, so that further rearing needs to be carried out in optimal environmental conditions, until crablets reach a larger size before being stocked to a pond. This study observed the behavioral thermoregulation of crablet instar phases, and determined the effect of several levels of water temperature on growth, survival, molting cycle, gill condition, and sex ratio in the nursery phase of *S. paramamosain*.

## 2. Materials and Methods

### 2.1. Experimental Production of Animals

Samples of mud crab megalopa (*S. paramamosain*) in this experiment were produced at the Institute of Tropical Aquaculture and Fisheries, Universiti Malaysia Terengganu hatchery, using local female broodstock from Setiu Wetland, Terengganu, Malaysia. Larval production started from broodstock maintenance, through spawning, incubation, and larval rearing. Female broodstock of mean BW (bodyweight) 348 ± 70.88 g, and mean CW/CL (carapace width/carapace length) 13.33 ± 0.65/8.83 ± 0.61 mm, were maintained in a 7.5 m^2^ square fiberglass tank, at a density of about 2 ind/m^2^. Broodstock were fed with Scad fish, *Decapterus macrosoma*, and occasionally (2–4 times a month) with shellfish (e.g., blood cockle, *Anadara granosa*, or mud clam, *Polymesoda erosa*), as often as once a day, at a dose of about 3% of biomass. Spawned females were allowed to remain in the broodstock tank until eggs started to turn black (estimated to hatch in 2–3 days), then were transferred to the incubation tank. The incubation tank was a 500 L round fiberglass tank (d = 1.1 m). After the eggs had hatched completely, aeration in the incubation tank was turned off for several minutes, then larvae that had clustered and actively swam on the surface were collected by gentle scooping.

Zoea 1 larvae were maintained in larval rearing tanks (500&1000 L round fiberglass tanks) at a density >100 ind/L. Larvae were fed with *Artemia* nauplii two to three times a day (morning, afternoon, and evening) with a density of about 2 to 5 ind/mL. In Zoea 1 and Zoea 2, larvae were given both live feed and frozen *Artemia* nauplii to facilitate feed capture. In the Zoea 3 to Megalopa (M) phase, larvae were only given live *Artemia* nauplii. Rearing time from Zoea 1 to M phase was 16 days, and on the 22nd day M was collected for experiment.

### 2.2. Thermal Preference

Thermal preference was determined by the acute method, with a horizontal gradient of temperature, using Crablet 1 (C1) stage. Crablets were acclimated at ambient temperature (27 to 30 °C), prior to assessment. Apparatus consisted of a PVC pipe 330 cm long, and 10.16 cm in diameter, with 21 virtual segments of 15 cm each. The depth of the water column was 8 cm, and a gradient was formed by placing one 300 W heater (Eheim^®^) at one end, whereas at the other end, extreme cold water was provided by adding frozen sea water (by plastic cup). The gradient in temperature ranged between 9 to 38 °C. In each virtual segment, a tube diffuser/aeration stone was placed along the gradient, to provide a very gentle aeration, and avoid stratification in the water column. The temperature was measured in each virtual segment with a glass thermometer. Crablets were not fed for 24 h before testing, to avoid interference from their diet. Within the formed gradient, one crablet was introduced at the virtual segment which had temperature as same as of the acclimation temperature [7]. The location of the organisms, and temperature of each segment were recorded every 10 min, for a total of 60 min. Although preference observations were done every 10 min, preferred temperature was only registered at the end of the time of exposure in the tube. Each individual was evaluated only once, and each experiment was carried out with three replications.

### 2.3. Critical Thermal Minimum (CTmin) and Critical Thermal Maximum (CTmax)

A 40-litre aquarium was used as a thermo-regulated bath, containing one experimental chamber (a plastic jar with 0.5 mm thickness with small holes in its sides). Both a glass thermometer, and DO (dissolved oxygen) meter (YSI^®^ professional series) were used to monitor temperature during the experiments.Two aeration stones placed in the aquarium to maintain oxygen saturation, and to avoid thermal stratification in the water column. A 1500 W immersion heater was placed in the bath for CTmax determination. To determine CTmin, this bath was conditioned as a cold chamber, using ice flakes. The heating and cooling rate were approximately 1 °C min^−1^. Visual monitoring was performed to describe the behavioral stress responses, shown by crablets exposed to increases and decreases in temperature. Observation of CTmax and CTmin ended when the crablets showed loss of righting position, i.e., when crablets were on their back and could not recover an upright posture (about 2 min). When the juveniles reached this point, they were returned to the acclimation temperature (ambient temperature, 27 to 30 °C) for a 96 h recovery period. Data of crablets that did not recover after 96 h was discarded. Determination of CTmin and CTmax of C1 (crablet instar) were performed three times each, with individual crablets used only once. A total of 40 and 35 crablets were used for CTmax and CTmin experiments, respectively. A thermal tolerance interval (TTI) was calculated as the difference between CTmax and CTmin mean values.

### 2.4. Nursery Experiment

A completely randomized design was used for this experiment, with four temperature condition treatments: water temperatures of 24, 28 and 32 °C, and ambient temperature (27 to 30 °C) as a control. The daily water temperature fluctuation of the 24, 28 and 32 °C treatments was <1 °C. Each treatment had three replicates, and each replication consisted of 10 samples. A Resun^®^ Chiller was used to control temperature in Treatments A (24 °C) and B (28 °C), while Treatment C (32 °C) used a 300 W (Eheim^®^) heater. Crablets were maintained individually in plastic cups with 6–9 cm of bottom diameter. The cups were placed in a rectangular fiber tank (117 cm length × 70 cm width) for each treatment, with approximately 7 cm of water depth. A recirculation aquaculture system (RAS) with gentle aeration was used to ensure mixing of the water bath. Water was exchanged every two days, as much as 20 to 30%. The used water salinity ranged from 25 to 30 ppt. During 45 days of nursery period, crablets were fed with frozen adult *Artemia* once a day (ad libitum). The observed parameters were growth performance, survival, molting cycle (molting interval in days, and molting increment,%, between crablet stages), and sex ratio. Based on the molting cycle data, the results from each crablet stage, in each experiment, were determined at the end of experiment. Carapace measurements were performed using digital calipers (0.01 mm), and weight measurements using a digital scale (0.01 g). Crablet stages, as described with C1, C2, C3 and so on, were determined based on molting activity observed daily [2,28].

Growth performance was evaluated using growth indices: specific growth rate (SGR) (% day^−1^), which was determined both in terms of BW (SGR_BW_) and CW (SGR_CW_), molting interval, and percentage of molt increment [29,30]. Besides that, survival rate, and sex ratio were calculated as follows:

- SGR_BW_ (% day^−1^) = ((ln final weight—ln initial weight)/days of culture) × 100
- SGR_CW_ (% day^−1^) = (ln final carapace width—ln initial carapace width)/days of culture × 100
- Percentage of molting increment (%) = ((postmolt CW—premolt CW)/premolt CW) × 100
- Molting interval (days) = time period between two molt events
- Survival rate (%) = (final amount of sample/initial amount of sample) × 100
- Sex ratio (%) = (male or female/(male + female)) × 100

### 2.5. Sexing of S. paramamosain Crablet

**Figure 1:**
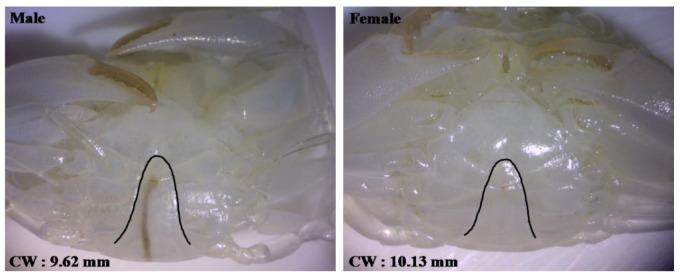
Estimation of male and female based on abdominal flap shape in Crablet 5 (C5) stages of mud crab.

Sexing of mud crab crablets was performed morphologically, through observation of the appearance of abdominal flaps, gonopods and gonophores [31,32]. Sexing was started in the Crablet 5 (C5) stage, where differences in abdominal flap began to appear more clearly under optical microscope (magnification of 8–20×). Female abdominal flaps in C5 stages were wider than male abdominal flaps, while the notch on the side for females was not as clear as for males (Figure 1).

### 2.6. Gill Histological Observation

Samples of C5 stage from each treatment were fixed in Davidson solution for 24–48 h, stored in a 70% ethanol solution until further normal histological processing. Parafin wax sections (5μm thick) were stained with H&E and were observed using Nikon^®^ microscope type Eclipse 80i, and image analysis (NIS-Elements D 2.30 227) software.

### 2.7. Data Analysis

Data of carapace size and ratio, survival, and molting interval were processed first with normality (Shapiro–Wilk test), and homogeneity test (Levene test). The data were transformed using square root transformation prior to analysis, if the original data did not meet standards of normality and homogeneity. Data were analyzed using analysis of variance (ANOVA), and post hoc Tukey’s test, to determine statistical significance. If the data did not meet parametric analysis standards, even though the data had been transformed, the data were then analyzed using non-parametric statistics, namely the Krusskal–Wallis ANOVA, and post hoc Dunn test. Statistical analyses were run on R-program, whereas tables and graphs were generated using Microsoft Excel program. Data of CTmax, CTmin, and preferred temperature, were presented as mean values with standard deviation.

## 3. Results

### 3.1. Thermoregulatory Behavior

**Figure 2:**
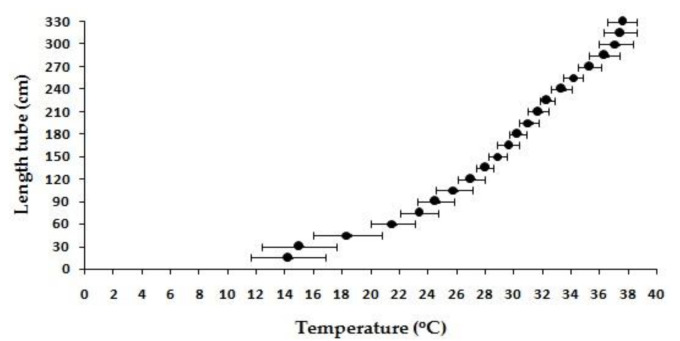
Temperature performance of the experimental devices used to evaluate preferred temperature.

The mean value of CTmin, CTmax, preferred temperature, and thermal tolerance interval (TTI) of C1, were 17.33 ± 0.58 °C, 40 ± 0.00 °C, 29.83 ± 2.47 °C, and 22.67 ± 0.58 °C, respectively, as listed in detail in Table 1. CTmin and CTmax values, obtained in the three replications, showed a narrow range for CTmin, and similar values for CTmax, while preferred temperature values showed a wide range. In the preferred temperature experiment, the temperature gradient in the apparatus had an interval of 14.2 to 37.56 °C (Figure 2).

### 3.2. Effect of Water Temperature on Growth Performance, Molting Cycle, Survival Rate, and Sex Ratio

**Figure 3:**
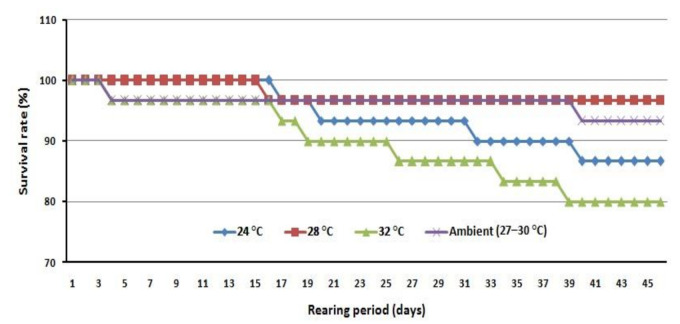
Survival rate daily during experiment.

Mean of final carapace dimension, for crablets kept at 28 °C, was significantly wider than for those kept at 24 °C (*p* < 0.05), but not significantly different than those kept at both 32 °C and ambient temperature. Means of CL and final BW were not significantly different (*p* > 0.05) between treatments, but 28 °C and ambient temperature treatment showed higher values than for both 24 and 32 °C treatments. SGR_BW_ observed for 24 °C was significantly different than for 28 °C (*p* < 0.05), but neither were significantly different from 32 °C, or ambient temperature. SGR_CW_ value observed for 24 °C was the lowest, and significantly different from both 28 °C and ambient temperature, but not significantly different from 32 °C. Highest survival rates were obtained at 28 °C, followed by ambient temperature, 24 °C, and 32 °C treatments, in order. Survival for 28 °C was significantly higher than 32 °C, but it was not significantly different from either 24 °C or ambient temperature. The daily survival rate in Figure 3 showed that 24 and 32 °C, which had the lowest survival rates, were gently decreased during the study. The stage composition of the mud crab crablets, at the conclusion of the experiment, showed that the 28 °C treatment had reached the C7 stage, whereas in the 32 °C and ambient temperature treatments only the C6 stage was reached. In the 24 °C treatment, C5 was the furthest crablet stage achieved. With respect to sex ratio, the 24 °C treatment showed highest male composition (*p* < 0.05) which was not significantly different from either of the 28 °C or ambient temperature treatments (Table 2).

**Figure 4:**
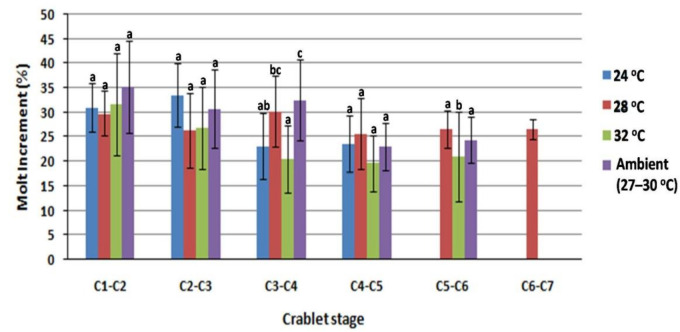
Molting increment in every stage of *S. paramamosain* crablet, during nursery phase, reared in different water temperatures (24, 28 and 32 °C) and ambient temperatures (27–30 °C). Means having different alphabet within treatment are significantly different (*p* < 0.05).

Molting interval (MI) mean was shortest (*p* < 0.05) between stages M to C5, and C1 to C5 for the 28 °C treatment, while it was longest for the 24 °C treatment (Table 3). MI was observed to be relatively longer as the stage increased, but fluctuations were found in the 28 °C and 32 °C treatments. Furthermore, molt increment tended to decrease as stage increased, in contrast to the molt interval. Molt increment in the earlier crablet stages (C1–C2,C2–C3, and C4–C5) were similar among treatments, but in both C3–C4 and C5–C6 intervals, crablets in both 28 °Cand ambient temperature showed significantly better performance (faster molt increment) (*p* < 0.05). Molt increment in 32 °C treatment showed the lowest value, particularly in the last three stages (C3 until °C6) (Figure 4). The carapace size of C4 and C5 was lowest for the 32 °C treatment (*p* < 0.05), eventhough the 32 °C treatment carapace had been of similar size to size in the other treatments during the C1–C3 stages (Table 4).

### 3.3. Gill Observation

**Figure 5:**
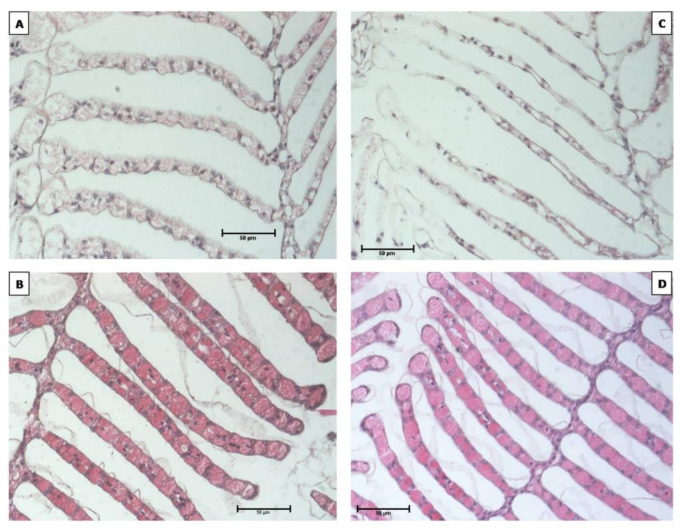
Condition of the gill lamella of *S. paramamosain* crablet, reared in different water temperature. (**A**) 24 °C, (**B**) 28 °C, (**C**) 32 °C, (**D**) ambient temperature (27–30 °C) (the scale length is 50 µm).

The color of the gill lamella at the treatment of low (24 °C) and high (32 °C) temperature treatments seemed paler, compared to the treatment temperature of 28 °C and ambient temperature (27–30 °C). In addition, the color, size, and shape of the gill lamella, at low and high temperature treatments also appeared different from both treatments 28 °C and ambient, while both the gill size and shape of treatments 28 °C and ambient were similar. The gill lamella size at high (Figure 5C) and low temperature (Figure 5A) treatment appeared thinner than for temperatures 28 °C and ambient, with thinnest gill size observedfor thehigh temperature treatment. The surface of the gill lamella at low temperatures appeared wrinkled and bumpy (Figure 5A).

## 4. Discussion

In this study, TTI of the crablet instar (C1) of *S. paramamosain* was 22.67 ± 0.58 °C (Table 1), which indicated that C1 of *S. paramamosain* has a wide temperature tolerance. Previous studies reported bigger TTI in other marine crabs, such as southern king crab, *Lithodes santolla*, between 20.5 to 25.5 °C [7], hairy-handed crab, *Hemigrapsus crenulatus*, between 27.19 to 29.86 °C [8], and blue swimming crab, *Portunus pelagicus*, between 24.99 to 26.04 °C [33]. These previous studies used older animal samples, so there is a possibility that the higher *S. paramamosain* crablet stages may also have a higher TTI value than the one reported in the current study. There was evidence that an increase in the amplitude of thermal tolerance occurred with advanced stages of the life cycle (juveniles), which demonstrated that thermal tolerance can change depending on age and life cycle stages [7,34,35].

TTI, and preferred temperature values of *S. paramamosain* C1, in this present study, showed that C1 had a wide temperature tolerance, making it suitable to be stocked into a safe, predator-free pond. Although survival rate of M to C1 was high in this study (>80%), stocking of M directly into a pond is not recommended [36], because at this phase the mud crabs are still considered very vulnerable. However, study on the viability of the stocking mud crab seed at lower stages than the crablet stage remains as a future challenge, since such practice could shorten larval rearing duration in the hatchery.

This study showed the effect of long-term exposure (chronic exposure) of water temperature on both growth, and survival rate of *S. Paramamosain*, during nursery phase. 28 °C treatment produced the highest mean of final CW, SGR_CW_, SGR_BW_, and survival rate (*p* < 0.05). The higher values of growth performance and survival rate at this temperature, compared to other treatments, showed that the optimal temperature for the nursery of *S*. *paramamosain* (started from M to several further crablet stages) was around 28 °C. The current findings are almost similar to the optimal temperature of 30 °C recommended for juveniles of *S. Serrata*, during nursery phase from C2 to C3 [28], and for other portunid species, such as chinese mitten crab, *Eriocheir sinensis*, for which temperatures of 28 and 30 °C were the optimal water temperatures for growth and molting in the juvenile phase [30].

Growth performance in this study, as measured by CW, and BW at harvest, MI (molting interval), molting increment, SGR in terms of BW and CW (SGR_BW_ and SGR_CW_), and stage composition, was influenced by different temperature regimes, where low (24 °C) and high temperatures (32 °C) tended to have lower values than for temperature regimes of both 28 °C and ambient temperature (27 to 30 °C). Temperature is one of the external factors that can affect the growth of crustaceans, where increasing temperature from optimal temperature can result in reduction of molt increment [37,38]. Compared to salinity, temperature is a more influential factor, that affects both survival and growth in juveniles of *S. serrata* [28], and the blue crab, *Callinectes sapidus* [39].

In this study, 32 °C treatment had the shortest MI (2.96 ± 1.22 days) compared to other treatments (*p* < 0.05), during the molting phase from C1 to C2, although its value was not significantly different from either the 28 °C or ambient temperature treatments. This result indicated that high temperature had an effect on accelerationof molting cycle at C1 to C2, but in subsequent phases, the 28 °C treatment had improved MI, even though the values were not significantly different from the 32 °C and ambient temperature treatments. Furthermore, MI from C5 to C6 for the32 °C treatment had the shortest MI, and its value was significantly different (*p* < 0.05) from both 28 °C and ambient temperature treatments. The shorter MI periods at high temperatures are common in crustaceans [30,38], but in this study MI fluctuations at 32 °C treatment (sometimes being the shortest and sometimes longest, compared with other treatments) provided an understanding that there may be other factors which accelerate molting significantly at high temperatures. Factors may include stable salinity, adequate water nutrient levels, or feed nutrition. Another important issue that requires attention is that, due to shorter MI at high temperatures, crablet size will be smaller at maturity stage, and this will affect individual reproductive output, and population in the wild [38].

In this study, the presence of daily salinity fluctuations (>1 ppt) at 32 °C, due to evaporation, may have had an effect on MI fluctuations at 32 °C. Future experiments need to minimize such salinity fluctuations for higher temperature treatments. Although it was known that mud crab have a broad tolerance to temperature and salinity [2,28,40,41], the variation in these two, environmental factors may have substantial effects on growth, survival, and production of crabs [28], and they need to be kept as stable as possible [42,43]. Salinity alone was reported to affect MI at the crablet stage. Gong et al. [2] reported that the longest MI of *S. Paramamosain*, in the C1 to C2 phase, was at salinity of 40 ppt, in comparison to salinities of 5, 10, 20, and 30 ppt (*p* < 0.05), which was thought to be related to a lower expression of Ecdysone receptor (Ecr) transcript in the crablet at salinity of 40 ppt. The results obtained by Ruscoe et al. [28] also showed that MI of *S. Serrata*, from C2 to C3, was longer at salinity 35 ppt in comparison to salinities of 5, 10, 20, and 30 ppt, at temperature conditions of 25, 30 and 35 °C.

Survival rate has been useful in identifying the effect of inadequate diets on the crab larvae [29,44], while it can also be an indicator of nonconformity of environmental conditions, as obtained in this study, where both highest (32 °C) and lowest (24 °C) temperatures exhibited lower survival. These results are similar to those of Gong et al. [2], and Ruscoeet al. [28], who studied effects of water temperature at C1 to C2 on *S. Paramamosain*, and at C2 to C3 of *S. Serrata*, respectively. In the current study, exposure of juveniles of *S. Paramamosain* at non-optimal temperatures for long-term, likely caused loss ofenergy, and triggered mortality. Optimization of physiological and biochemical processes that provide maximal efficiency in energy use can occur within the optimal temperature range [11,12], while slower growth and lower survival rate result below or above theoptimal temperature range [45]. Paital andChainy [46] supposed that fluctuation on either side of ambient habitat temperature may adversely influence mitochondrial respiration, and oxidative metabolism, in *S. serrata.* Further, Matozzo et al. [47] concluded that temperature influenced crab biological responses, and indicated that mediterranean shore crab, *Carcinus aestuarii*, modulated its cellular and biochemical parameters (mainly haemocyte proliferation, cell-free haemolymph protein concentrations, and cell-free haemolymph phenoloxidase activity) in order to cope with temperature. In the crablet phase, *S. paramamosain* had a better resistance to low temperature, which could be attributed to the increasing thickness of the integument with growth, that provides increasing protective, mechanical protection against abrupt changes in temperature [41]. Gong et al. [2] reported that the percentage of molting success of *S. Paramamosain* can reach 86.7%, from C1 to C2, under water temperature of 20 °C (salinity of22 ± 1 ppt), but at water temperature of 14 °C, C1 becomes inactive with a substantial decrease in feed consumption, and C1 also did not molt to C2, during the current study. In C2 to C3 phase, Ruscoe et al. [28] found that, at water temperature of 20 °C, *S. serrata* could only molt as much as 10 and 20%, at salinity conditions of 5 and 20 ppt, respectively. Generally, the optimal temperature range for tropical and subtropical crab species is above 25 °C. Temperatures below 20 °C cause increase in larval mortality [48], particularly due to decline in feeding at low temperature [28,41].

Histological observationof the gill lamella section showed that various temperature treatments had a direct effect on the gills of *S. paramamosain* crablets. Although the reason for changes in the size of gill lamella remain unknownin this study, they are likely to be either a form of adaptation to temperature changes, or to result from negative impact of long-term exposure to temperatures above or below the optimal temperature. For example, one of the ways in which intertidal decapod crustaceans adapt to an environment, which is sometimes without water, is the specialization of the structure of their branchia apparatus, which functions to prevent the gill lamella from adhering together, and reduces availablesurface area for gas exchange [49]. The change of the gill lamella size confirms the negative impact of non-optimal culture conditions on aquatic organisms, especially in long-term exposure to extreme water temperature. Such information is fundamentally important to understanding of response and adaptation mechanisms of crabs, in particular the response of gills to environmental change with respect to morphological and physiological effects [50]. The observation confirms that gillcondition is an efficient biomarker for water quality studies [51], where crustacean gills mediating exchange processes between the individual and its ambient medium, perform complexmetabolic roles, including uptake of oxygen, excretion of carbondioxide, active ion transport in osmoregulating species, acid-base balance of the hemolymph, and excretion of nitrogenous end products [52].

Male ratio was higher at low temperature treatment (24 °C) than for the other temperature treatments (*p* < 0.05), which indicated a possibility of water temperature having an affect on sex ratio in mud crab, such as has been commonly reported in teleostei fish [19,20,21,22,23,24], and also in some crustaceans [18,25,26]. Interestingly, male ratio produced in this study was higher at low temperature (24 °C), whereas in teleostei fish, high temperature of ≥28 °C and aboveusually produces higher male ratio [19,20,21]. Nevertheless, there are also fish species which have more male composition at lower temperature, and even some species of fish that have higher male ratio at extreme (low and high) temperatures [27].

The presence of sex reversal in *S. paramamosain* needs to be proven using genetic assessment, and at present it can be done using the method that has been introduced by Shi et al. [53], starting from the M stage. Masculinization of mud crab has previously been successfully carried out by injecting androgenic gland hormone into newly developed females, which was claimed as the first report of sex reversal induced by injection of androgenic gland extract in brachyuran crabs [54]. On that basis, there is the possibility that another treatment commonly used in fish for masculinization, could also be applied to the mud crab, i.e., temperature. Besides the possibility of producing sex reversal in mud crab through temperature treatment, there is also the possibility that water temperature could accelerate appearance of abdominal flap differentiation, for both male and female. In this present study, the shape of abdominal flap observed as male at 24 °C treatment looked clearer (more pointed) than the male abdominal flap of crabs at the same stage in other treatments.

Generally, our findings showed that long-term exposure at various water temperature treatments can be used to determine the optimal water temperature, similar to how short exposure treatment can be used to determine preferred temperature. Chronic exposure to thermal stress allows determination of its effects on larval growth and survival [55], and on the levels of tolerance, behavior, and physiological effects [56]. Study of both chronic and acute thermal tolerance of intertidal biota is still lacking [57]. Knowledge of effects and tolerances needs to be continuously improved, in order to quantify the impact of global warming on various aquatic species in the future, with particular relevance to aquaculture development and its sustainability.

## 5. Conclusions

TTI and preferred temperature values observed in the current study showed that C1 of *S. paramamosain* had a wide temperature tolerance with CTmin and CTmax values of 17.33 ± SD 0.58 °C and 40 ± SD 0.00 °C, respectively. The optimal temperature range that was suggested for long-term nurseryphase (started from M stage) of *S.* *paramamosain* was 28 to 30 °C. Below or above optimal temperature range could have negative impacts on growth performance, survival, or gill lamella structure of *S. paramamosain* crablet during long-term nursery phase. Findings of this study could increase crablet hatchery productivity, and reduce dependence on seeds taken from the wild. In addition, the potential of monosex seed production of *S. paramamosain* through temperature treatment needs to be studied further in future.

## Figures and Tables

**Table 1:** Critical thermal limits and preferred temperature values of crablet instar of mud crab (*S. paramamosain*).

Experiment	CTmin (°C)	CTmax (°C) **	Preferred Temperature (°C)	* Thermal Tolerance Interval (TTI)
1	17	40	27	23
2	18	40	31	22
3	17	40	31.5	23
Average (±SD)	17.33 ± 0.58	40 ± 0.00	29.83 ± 2.47	22.67 ± 0.58

* TTI was calculated as the difference between CTmin and CTmax mean values. ** Oxygen level was >4 ppm.

**Table 2:** Growth performance, survival, and sex ratio of *S. Paramamosain*,during nursery phase, reared in different water temperatures (24, 28 and 32 °C and ambient temperatures (27–30 °C)).

Parameter	Treatment *			
	A (24 °C) ± SD	B (28 °C) ± SD	C (32 °C) ± SD	D (Ambient Temp.) ± SD
Experimental duration (days)	45	45	45	45
Initial animal sample (ind.)	30	30	30	30
Final animal sample (ind.)	26	29	24	28
Mean of initial carapace width (mm)	1.62 ± 0.05	1.62 ± 0.05	1.62 ± 0.05	1.62 ± 0.05
Mean of final carapace width (mm)	8.81 ± 1.29 ^a^	12.30 ± 1.52 ^b^	9.59 ± 0.68 ^ab^	11.73 ± 1.65 ^ab^
Mean of final carapace length (mm)	6.27 ± 0.93 ^a^	8.38 ± 0.89 ^a^	6.76 ± 0.53 ^a^	8.26 ± 1.06 ^a^
Mean of initial body weight (g)	0.0035 ± 0.00	0.0035 ± 0.00	0.0035 ± 0.00	0.0035 ± 0.00
Mean of final body weight (g)	0.11 ± 0.05 ^a^	0.29 ± 0.09 ^a^	0.12 ± 0.03 ^a^	0.27 ± 0.09 ^a^
Specific growth rate (SGR_BW_) (%)	7.28 ± 1.31 ^a^	9.69 ± 0.75 ^b^	7.83 ± 0.56 ^ab^	9.48 ± 1.02 ^ab^
Specific growth rate (SGR_CW_) (%)	3.74 ± 0.34 ^a^	4.50 ± 0.28 ^b^	3.95 ± 0.16 ^a^	4.38 ± 0.35 ^b^
Composition of C4 (%)	30.55 ± 4.81	-	-	-
Composition of C5 (%)	69.44 ± 4.81	3.33 ± 5.77	6.66 ± 11.54	8.93 ± 7.79
Composition of C6 (%)	-	74.40 ± 21.15	93.33 ± 11.54	91.07 ± 7.79
Composition of C7 (%)	-	22.27 ± 15.38	-	-
Survival Rate (%)	87 ± 5.77 ^ab^	97 ± 5.77 ^a^	80 ± 5.00 ^b^	93 ± 5.77 ^ab^
Sex ratio (%)				
Male	80.09 ± 18.86 ^a^	44.81 ± 10.50 ^ab^	41.94 ± 19.44 ^b^	76.30 ± 5.13 ^ab^
Female	19.91 ± 18.86 ^a^	55.18 ± 10.50 ^ab^	58.05 ± 19.44 ^b^	23.70 ± 5.13 ^ab^

* Means having different superscripts (alphabetical) within row are significantly different (*p* < 0.05). SGR_BW_ = specific growth rate in terms of body weight, SGR_CW_ = specific growth rate in terms of carapace width. C4 = Crablet 4 stage, C5 = Crablet 5 stage, C6 = Crablet 6 stage, and C7 = Crablet 7 stage.

**Table 3:** Molting interval of *S. Paramamosain*, during nursery phase, reared in different water temperatures (24, 28 and 32 °C and ambient temperatures (27–30 °C)).

**Parameter**	**Molting Interval (MI) (days)**			
	**24 °C**	**28 °C**	**32 °C**	**27–30 °C**
M * to C1	1.46 ± 0.72 ^a^	1.80 ± 0.74 ^a^	1.63 ± 0.56 ^a^	1.33 ± 0.51 ^a^
C1 to C2	7.63 ± 1.25 ^a^	3.92 ± 1.86 ^ab^	2.96 ± 1.22 ^b^	4.67 ± 0.50 ^ab^
C2 to C3	8.78 ± 1.76 ^a^	5.68 ± 1.37 ^b^	5.77 ± 0.87 ^b^	5.88 ± 0.99 ^b^
C3 to C4	10.83 ± 3.60 ^a^	6.62 ± 0.99 ^b^	7.37 ± 1.78 ^ab^	7.34 ± 1.03 ^ab^
C4 to C5	13.00 ± 1.52 ^a^	8.47 ± 1.64 ^b^	10.50 ± 1.29 ^b^	9.33 ± 2.18 ^b^
C5 to C6	-	10.66 ± 2.03 ^a^	7.66 ± 0.92 ^b^	11.95 ± 2.14 ^a^
C6 to C7	-	9.25 ± 0.66	-	-
Mean duration from M to C5 stage (days)	38.58 ± 2.90 ^a^	27.03 ± 0.89 ^b^	29.68 ± 0.91 ^ab^	28.18 ± 3.40 ^ab^

* Megalopa’s age when they stocked (±6 days). Means having different superscripts (alphabetical) within row are significantly different (*p* < 0.05). M = Megalopa stage, C1 = Crablet 1 stage, C2 = Crablet 2 stage, C3 = Crablet 3 stage, C4 = Crablet 4 stage, C5 = Crablet 5 stage, C6 = Crablet 6 stageand C7 = Crablet 7 stage.

**Table 4:** Mean carapace sizes (carapace width and carapace length) of *S.paramamosain* in different stage during nursery phase reared in different water temperatures (24, 28 and 32 °C) and ambient temperatures (27–30 °C).

Parameter	Treatment			
	24 °C	28 °C	32 °C	27–30 °C
Carapace Width:				
M	1.62 ± 0.05	1.62 ± 0.05	1.62 ± 0.05	1.62 ± 0.05
C1	3.35 ± 0.13 ^a^	3.40 ± 0.48 ^a^	3.46 ± 0.18 ^a^	3.41 ± 0.10 ^a^
C2	4.43 ± 0.17 ^a^	4.50 ± 0.14 ^ab^	4.50 ± 0.19 ^ab^	4.80 ± 0.34 ^b^
C3	5.89 ± 0.41 ^a^	5.62 ± 0.42 ^a^	5.73 ± 0.24 ^a^	6.00 ± 0.25 ^a^
C4	7.47 ± 0.60 ^ab^	7.35 ± 0.56 ^ab^	7.07 ± 0.56 ^a^	7.83 ± 0.61 ^b^
C5	9.48 ± 0.70 ^a^	9.12 ± 0.68 ^a^	8.22 ± 0.60 ^b^	9.56 ± 0.73 ^a^
C6	-	11.85 ± 0.85 ^a^	9.63 ± 0.37 ^a^	12.08 ± 1.07 ^a^
C7	-	14.30 ± 0.72	-	-
Carapace Length:				
C1	3.02 ± 0.10 ^a^	3.00 ± 0.18 ^a^	3.17 ± 0.16 ^a^	3.03 ± 0.10 ^a^
C2	3.64 ± 0.12 ^a^	3.53 ± 0.18 ^a^	3.62 ± 0.20 ^a^	3.71 ± 0.32 ^a^
C3	4.43 ± 0.20 ^a^	4.33 ± 0.30 ^a^	4.43 ± 0.21 ^a^	4.55 ± 0.30 ^a^
C4	5.33 ± 0.40 ^ab^	5.39 ± 0.34 ^ab^	5.09 ± 0.46 ^a^	5.69 ± 0.40 ^b^
C5	6.75 ± 0.50 ^a^	6.46 ± 0.43 ^a^	5.85 ± 0.42 ^b^	6.78 ± 0.46 ^a^
C6	-	8.16 ± 0.51 ^ab^	6.78 ± 0.23 ^a^	8.49 ± 0.69 ^a^
C7	-	9.31 ± 0.41	-	-

Means having different superscripts within row are significantly different (*p* < 0.05).

## Data Availability

The data presented in this study are available on request from the corresponding author.
